# Direct hepatic tissue PO_2 _measurements in sepsis and tamponade models

**DOI:** 10.1186/cc10151

**Published:** 2011-06-22

**Authors:** E Silva, P Rehder, AJ Pereira, F Colombari, LFP Figueiredo

**Affiliations:** 1 Institute of Heart, University of São Paulo, São Paulo - SP, Brazil

## Introduction

Tissue hypoxia diagnosis at the bedside remains a huge challenge for intensivists, and surrogate markers of tissue oxygen utilization are used instead. The precise correlation between them is not well defined.

## Objective

To verify the correlation between portal blood flow, O_2 _and CO_2 _gradients, hepatic lactate gradient with hepatic tissue PO_2_.

## Methods

This is an observational experimental study, in which 16 large, male, white pigs, about 35 kg, were allocated into two groups: sepsis (*n *= 8), and tamponade (*n *= 8). All protocols were approved by the institutional review board for animal experiments. Anesthesia: premedication with intramuscular ketamine (10 mg/kg) plus midazolam (0.25 mg/kg); induction with intravenous propofol 5 mg/kg (at maximum) followed by continuous isoflurane (1.5%), fentanil 2.5 μg/kg/hour and pancuronium 0.24 mg/kg/hour. Mechanical ventilation settings: Vt 10 ml/kg, PEEP 5 cmH_2_O, respiratory rate set to normocapnia and FiO_2 _adjusted to arterial oxygen partial pressure 60 to 100 mmHg. Continuous gas analysis was also performed. Electrocardiography, invasive pressure in dissected femoral artery, right atria and ventricular pressures after left internal jugular dissection; etCO_2 _(by gas analyzer), pulmonary artery catheter, portal vein flow Doppler ultrasound, and small bowel tonometry, after median laparotomy. Liver tissue pO_2 _monitoring: pO_2 _- fluorescence quenching optode - and LDF - laser Doppler fluxometry - probes were directly inserted inside liver parenchyma (Oxford-Optronix, UK). Other procedures: cistostomy (to monitor diuresis), inferior vena cava (by femoral) and superior vena cava (by right jugular) vein catheterizations. Portal vein catheter, after liver hilus dissection (Seldinger) and fluoroscopy-guided right suprahepatic vein catheterization. After experiments, pigs were sacrificed with sedative overdose and 20 ml KCl 19.1% injection. Sepsis was induced by spread of 150 ml warm saline diluted 1 g/kg feces in the peritoneal cavity. Tamponade: mini-thoracotomy and a mono-lumen intrapericardium catheter positioning to arouse cardiac tamponade, targeting 20% of baseline decrease in cardiac output at each time phase. Data were analyzed in Excel 2007.

## Results

**Figure 1:**
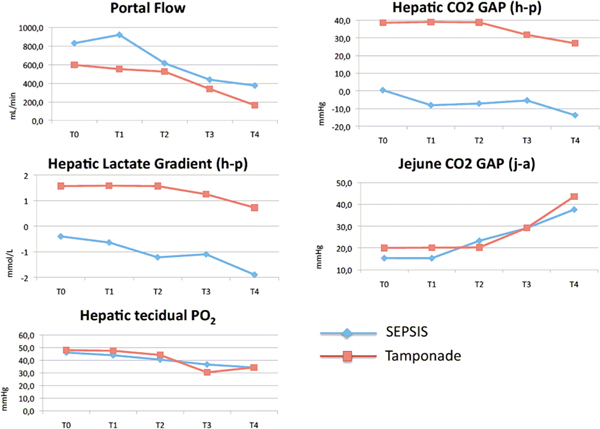
**Portal blood flow, hepatic CO_2 _gap, hepatic lactate gradient, jejune CO_2 _gap and hepatic tissue PO_2 _over time in both groups, sepsis and tamponade**.

In both groups, there was a progressive decrease in portal blood flow, an increase in jejune-portal CO_2 _gap, and a decrease in hepatic tissue PO_2_. Interestingly, there was a progressive hepatic lactate consumption as hepatic tissue PO_2 _decreases. Figure 1 (overleaf) depicts the behavior of the above variables.

## Conclusion

Hepatic tissue PO_2 _paralleled portal blood flow and was inversely related to the jejune tissue PCO_2 _gap. Liver has increased lactate consumption as hepatic tissue PO_2 _decreased.

